# The Barrow Biomimetic Spine: Comparative Testing of a 3D-Printed L4-L5 Schwab Grade 2 Osteotomy Model to a Cadaveric Model

**DOI:** 10.7759/cureus.2491

**Published:** 2018-04-17

**Authors:** Michael A Bohl, Michael A Mooney, Garrett J Repp, Claudio Cavallo, Peter Nakaji, Steve W Chang, Jay D Turner, U. Kumar Kakarla

**Affiliations:** 1 Department of Neurosurgery, Barrow Neurological Institute, St. Joseph's Hospital and Medical Center, Phoenix, USA; 2 Biomedical Engineering, Barrow Neurological Institute, St. Joseph's Hospital and Medical Center, Phoenix, USA

**Keywords:** schwab grade 2 osteotomy, spinal deformity, 3d-printed spine model

## Abstract

Introduction

The Barrow Biomimetic Spine project is an ongoing effort to develop a three-dimensional (3D)-printed synthetic spine model with high anatomical and biomechanical fidelity to human tissue. The purpose of this study was to evaluate the biomechanical performance of an L4-L5 3D-printed synthetic spine model in a lordotic correction test after Schwab grade 2 osteotomies as compared to human cadaveric spines that have undergone the same osteotomies and lordotic correction.

Methods

Ten different L4-L5 synthetic spine models were 3D printed. Each print varied in either the material used for the soft tissue components, the infill density of the bony and soft tissue structures, the pre-correction disc height, or the model orientation on the print bed. Each print was instrumented with pedicle screws and underwent a Schwab grade 2 osteotomy. Changes in disc height measurements and end-plate angle were compared against cadaveric data acquired using the same study method.

Results

A simple linear correlation analysis demonstrated that for horizontally printed models using PolyFlex (Polymaker, New York, NY, USA)(models 1-3, 8, 10), the pre-correction posterior disc height and lordotic correction were moderately correlated (r = 0.56), but this correlation did not achieve statistical significance (P = 0.12). Regression analysis demonstrated a very strong correlation between lordotic correction and change in posterior disc height (r = 0.92, P < 0.001). Models printed either vertically (models 4-6) or with low bone density and high soft tissue density (model 10) appeared to perform the most similarly to the cadaveric tissue.

Discussion

The 3D-printed synthetic spine models demonstrated predictable and reliable performance in a lordotic correction test based on their respective material qualities and print densities. The print variables tested further demonstrated that this model is capable of achieving high biomechanical fidelity to cadaveric tissue when subjected to the same lordotic correction test after Schwab grade 2 osteotomies.

## Introduction

Three-dimensional (3D) printing is an additive manufacturing technology with tremendous potential in the field of spine surgery. Patient-specific 3D-printed spine models are now regularly used at some institutions for preoperative planning and intraoperative reference, and have reportedly been used as patient-specific implants for complex spinal reconstructions, patient consent and resident educational models, and scaffolds for the laboratory regeneration of disc tissue [1-6]. A field that has yet to receive as much attention in the literature, but for which 3D-printing technology holds great potential, is the field of spine biomechanical research. Researchers studying the biomechanical properties of the spine have traditionally relied on cadaveric tissue, computerized models, or “block-and-spring” models for data generation and testing [7-11]. Each of these models comes with significant limitations, including high inter-specimen variability and limited shelf life for cadaveric tissue, questionable validity and inability to perform physical testing with computerized models, and poor anatomical fidelity and variable biomechanical performance of block-and-spring models. To overcome these limitations, attempts have been made to create a synthetic spine model with high anatomical and biomechanical fidelity to human tissue [11-13]. Early results with these models are promising, but several significant limitations remain, including high cost, long production time, and the inability to customize the models for testing specific anatomical or pathological states.

The Barrow Biomimetic Spine project is an ongoing effort to develop a 3D-printed synthetic spine model with high anatomical and biomechanical fidelity to human tissue that is also completely customizable to any given anatomical or pathological state. Yet-to-be-published data from the authors’ laboratory have demonstrated this model’s high anatomical and radiographic fidelity to human tissue [unpublished data], as well as the high biomechanical fidelity of the model’s bony structures [unpublished data]. However, no studies have yet validated the biomechanical performance of this model’s soft tissue structures. The disc and ligamentous support structures of the human spine are complex and, compared to bony tissue, are much more difficult to recreate with synthetic materials. As such, we have taken a stepwise approach to validating the biomechanical performance of these various structures, beginning first with a model that contains only the vertebral bodies, interbody disc, anterior longitudinal ligament, and posterior longitudinal ligament. This model effectively recreates a surgical procedure in which all the ligamentous support structures of the posterior column are removed: the Schwab grade 2 osteotomy [14]. The purpose of this study was to evaluate the biomechanical performance of an L4-L5 3D-printed synthetic spine model in a lordotic correction test after a Schwab grade 2 osteotomy as compared to human cadaveric spines that have undergone the same osteotomies and lordotic correction.

## Materials and methods

Creation of spine models

A high-resolution computed tomographic (CT) scan of a normal spine was segmented using Materialise Mimics software (Materialise, Plymouth, Michigan, USA). Bony and soft tissue anatomical data were extracted and converted to the stereolithography (.stl) file format. The .stl files of discreet anatomical components of the spine (L4 and L5 vertebral bodies, L4-5 disc, and anterior and posterior longitudinal ligaments) were imported into the Simplify3D software platform (Simplify3D, Blue Ash, Ohio, USA), where they were then reassembled in the correct anatomical configuration. A FlashForge Creator Pro with dual extruders (FlashForge, City of Industry, CA, USA) was used for the creation of all the models tested in this study.

Study design

**Figure 1:**
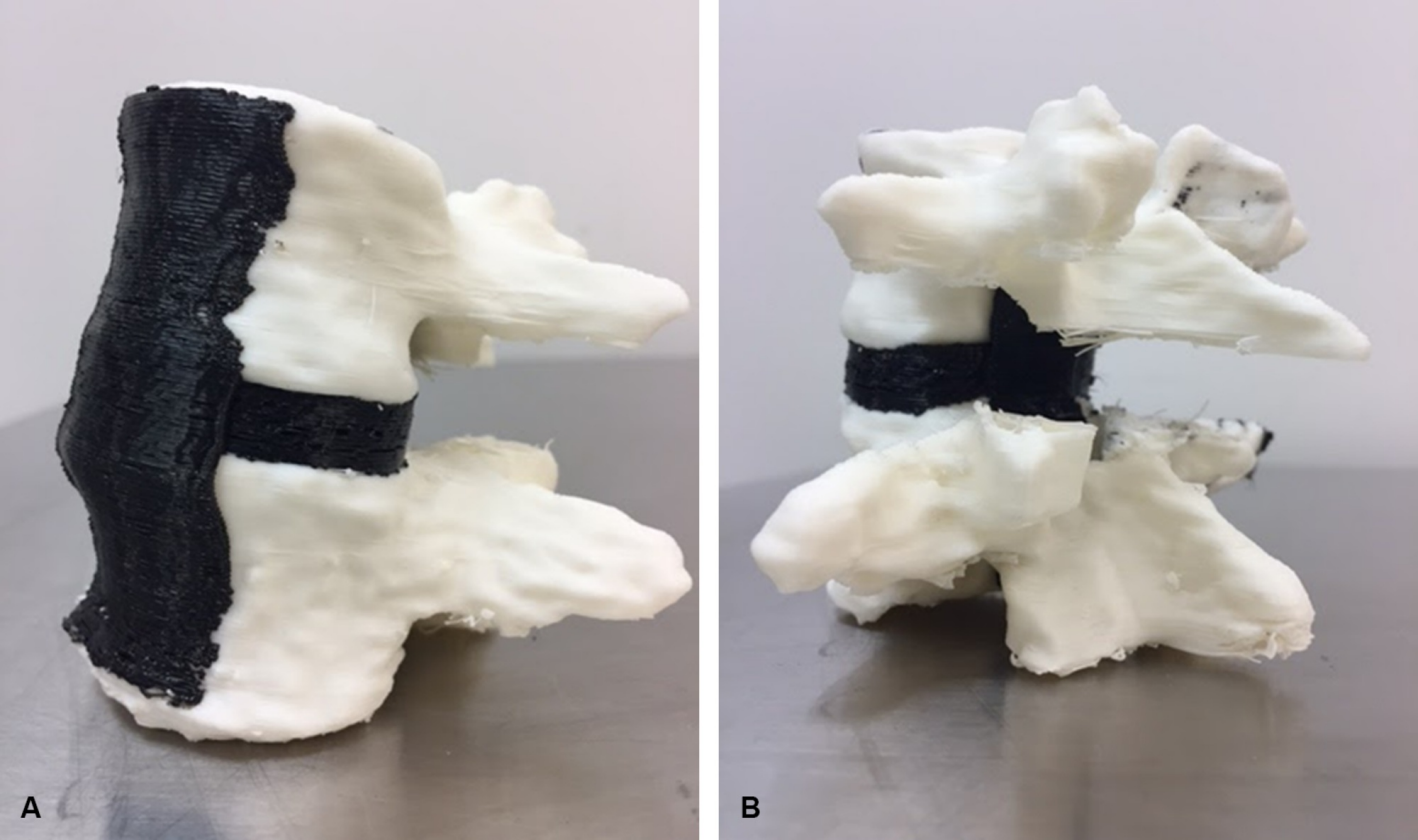
Photographs of model 1 from (A) an anterior oblique view and (B) a posterior oblique view. Note the synthetic bone in white, and the soft tissue structures (anterior and posterior longitudinal ligaments, and intervertebral disc) in black*. Used with permission from Barrow Neurological Institute, Phoenix, Arizona.*

**Figure 2:**
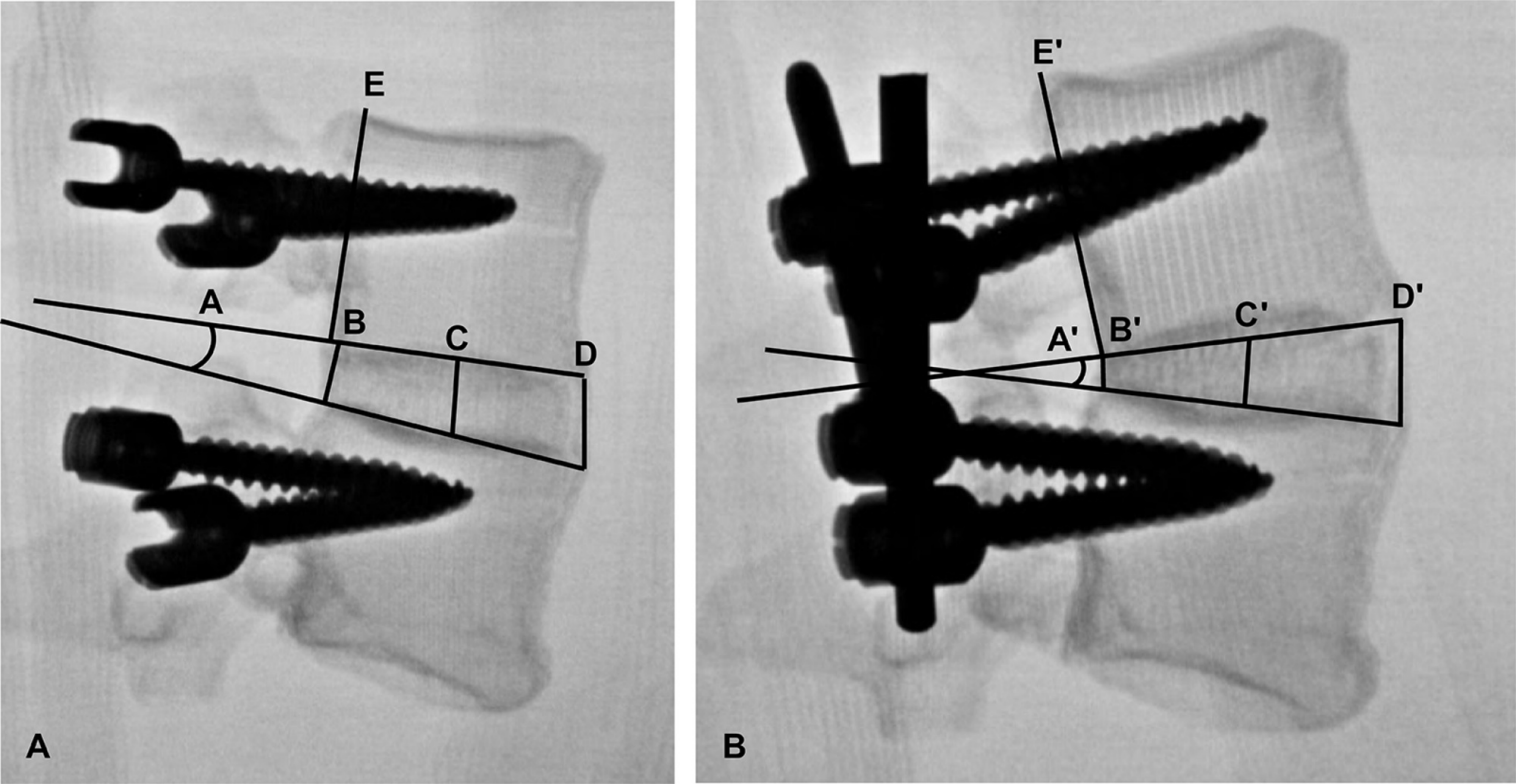
Lateral fluoroscopic views of a synthetic spine model (A) before and (B) after lordotic correction. A = pre-correction lordosis, B = pre-correction posterior disc height, C= pre-correction middle disc height, D = pre-correction anterior disc height. E = pre-correction superior vertebral body height. A'-E' = post-correction measurements. *Used with permission from Barrow Neurological Institute, Phoenix, Arizona.*

Ten different L4-L5 synthetic spine models were 3D printed. Each print varied in either the material used for the soft tissue components, the infill density of the bony and soft tissue structures, the pre-correction disc height, or the model orientation on the print bed. See Table 1 for the specific variables used to print each of the 10 spine models. Print variables held constant across all 10 models included the material used for the vertebral bodies (acrylonitrile butadiene styrene), as well as the number of shells used for both the vertebral bodies and soft tissue structures (four shells for all 10 models). Once the models were printed, they underwent Schwab grade 2 osteotomies, which included complete resection of the superior and inferior articulating processes of the bilateral facet joints at the L4-L5 level so that no bone was present between the L4 and L5 pedicles. In humans, Schwab grade 2 osteotomies also include resection of the supraspinous ligament, interspinous ligament, and ligamentum flavum. This technique is believed to destabilize the posterior column sufficiently to achieve 5-10 degrees of lordotic correction with posterior column compression. The complete facetectomy also decompresses the neural foramina adequately to allow for posterior column compression without injury to the exiting nerve root. Each model was then instrumented with 7.5-mm × 45-mm pedicle screws. See Figures 1A-1B for photographs of model 1 after Schwab grade 2 osteotomies. Lateral fluoroscopic films were then obtained of the models before and after lordotic correction (Figures 2A-2B). The maximum lordotic correction was determined as the point at which further compression of the pedicle screws began to result in pedicle screw failure or the fracture of pedicles.

**Table 1:** Variables for the synthetic spine models Abbreviations: PDH, posterior disc height, TPU, thermoplastic polyurethane; S-PLA, soft polylactic acid *These values are a normalized ratio of PDH to the height of the superior vertebral body and are, therefore, unitless. This was done to account for errors introduced by variabilities in fluoroscopic magnification.

Model No.	Soft Tissue Material	Density of Vertebral Bodies	Density of Soft Tissue	Print Orientation	Pre-correction PDH (normalized ratio)*
1	PolyFlex	10%	10%	Horizontal	0.158
2	PolyFlex	20%	20%	Horizontal	0.165
3	PolyFlex	30%	30%	Horizontal	0.170
4	PolyFlex	10%	10%	Vertical	0.167
5	PolyFlex	20%	20%	Vertical	0.153
6	PolyFlex	30%	30%	Vertical	0.153
7	S-PLA	20%	20%	Horizontal	0.123
8	PolyFlex	30%	10%	Horizontal	0.113
9	TPU	20%	20%	Horizontal	0.110
10	PolyFlex	10%	30%	Horizontal	0.139

The results from the synthetic L4-L5 models were then compared against the results of a cadaveric study performed by the same lead authors using the same osteotomy and lordotic correction techniques [unpublished data]. As was done in the cadaveric study, pre- and post-correction vertebral end-plate angles and disc heights were measured. The anterior (ADH), middle (MDH), and posterior disc heights (PDH) were measured. Figure 2 demonstrates the lateral fluoroscopic radiographs taken of the models before and after lordotic correction, as well as the measurements taken in each radiograph. Average disc height was calculated as the mean of the ADH, MDH, and PDH. All end-plate angles and disc height measurements were taken three times and averaged to minimize the risk of measurement errors. Disc height measurements were normalized to the L4 vertebral body height to account for variations in fluoroscopic magnification between different radiographs.

Statistical analyses included the Shapiro-Wilk test for normality of the data, as well as a simple linear correlation and regression analysis to determine the correlation between pre-correction PDH and lordotic correction achieved for a subset of similarly printed models, specifically the horizontally printed models made with PolyFlex (Polymaker, New York, NY, USA) (models 1-3, 8, 10). Simple linear correlation and regression analyses were also performed to compare the correlation between the lordotic correction achieved and the change in disc height (PDH, MDH, and ADH) measurements after correction. Because of the small sample size, a P value of < 0.10 was considered significant. This higher P value cutoff for significance increases the risk of type 1 errors; however, with a sample size of only 10, statistical significance at a P value of 0.05 or less would require very large differences between the study groups. Moderate to small differences can be equally important when measuring disc heights and vertebral end-plate angles in the lumbar spine, as small changes in lumbar lordosis can result in large changes to overall spinal balance. We therefore decided that increasing the maximum P value for significance would be worth the increased risk of type 1 errors. Descriptive statistics are reported for the comparison of synthetic models. With the linear regression equation generated from a comparison cadaveric study [unpublished data], lordotic corrections for each of the 10 synthetic models were predicted and compared against the actual correction achieved. The percent error for each of the models was then calculated to determine which models performed most similarly to the reference cadaveric tissue [unpublished data].

## Results

**Figure 3:**
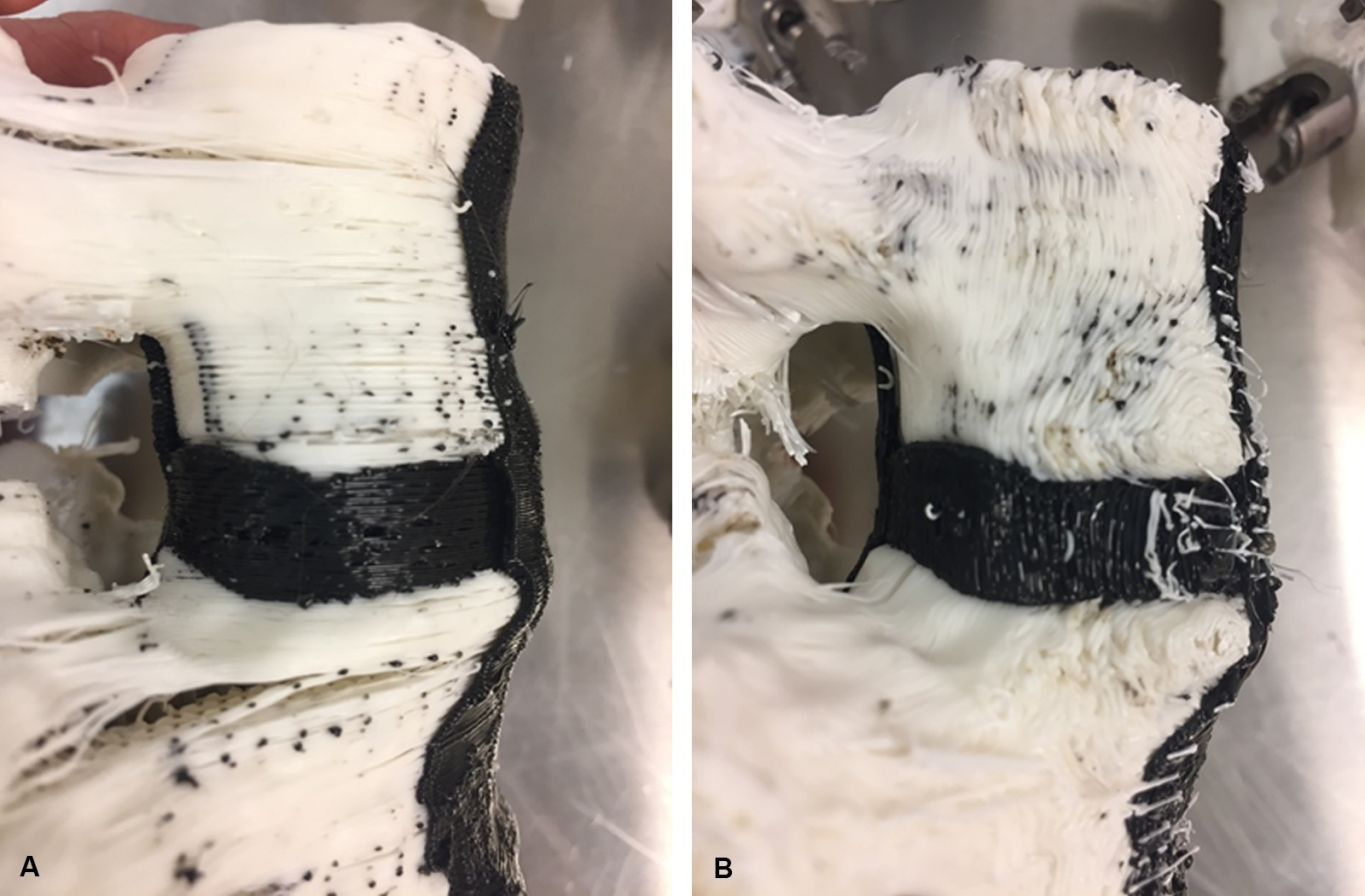
Photographs demonstrating a lateral view of (A) a horizontally printed model and (B) a vertically printed model. Note the difference in orientation of the layers of plastic filament that comprise the models. *Used with permission from Barrow Neurological Institute, Phoenix, Arizona.*

All 10 synthetic spine models were successfully printed and underwent lordotic correction after Schwab grade 2 osteotomies. Table 2 summarizes the comparative analyses of the 10 synthetic models. The models that performed most closely to the cadaveric data were models 5 and 10. It appeared that print orientation had an effect on biomechanical performance and achievable lordotic correction, with vertically printed models having a mean standard error of 31% (models 4-6), compared to a mean standard error of 60% for horizontally printed models with the same pre-correction PDH (models 1-3). Figures 3A-3B show the difference between a horizontally and vertically printed model, specifically the difference in orientation of the plastic layers used to build the models.

**Table 2:** Model performance Abbreviations: ADH, anterior disc height; AvDH, average disc height; MDH, middle disc height; PDH, posterior disc height *Positive values denote a reduction in disc height; negative values denote an increase in disc height. All values are the difference of two unitless ratios of disc height measurement to L4 vertebral body height measurement. This was done to correct for differences in fluoroscopic magnification. †Determined using linear regression equation from cadaveric data predicting lordotic correction using pre-correction PDH [unpublished data]. ‡Absolute value.

Model No.	Change in PDH*	Change in MDH*	Change in ADH*	Change in AvDH*	Lordotic Correction Achieved	Predicted Lordotic Correction†	Percent Error for Lordotic Correction‡
1	0.104	0.045	-0.014	0.045	8.11	5.37	51%
2	0.095	0.038	-0.079	0.018	8.55	5.98	43%
3	0.131	0.020	-0.073	0.026	11.87	6.39	86%
4	-0.014	-0.013	-0.058	-0.028	3.41	6.14	44%
5	0.014	0.024	-0.055	-0.006	4.36	5.02	13%
6	0.028	0.008	-0.037	0.000	3.17	4.94	36%
7	0.053	0.001	-0.082	-0.009	8.08	2.45	230%
8	0.050	-0.027	-0.080	-0.019	6.31	1.62	290%
9	-0.001	-0.023	-0.034	-0.019	0.63	1.44	56%
10	0.015	0.021	0.000	0.012	2.92	3.81	23%

The Shapiro-Wilk test was performed for the data on disc height and change in lordosis. The calculated P values ranged from 0.905 to 0.955. The data were therefore considered to be normally distributed. The simple linear correlation analysis demonstrated that for horizontally printed models using PolyFlex (models 1-3, 8, 10), the correlation was moderate (r = 0.56) between pre-correction PDH and lordotic correction, but this correlation did not achieve statistical significance (P = 0.12). Linear correlation and regression analysis demonstrated a very strong correlation between lordotic correction and change in PDH (r = 0.92, P < 0.001, F = 44.78, Y = 0.013X–0.028). ADH change was moderately, though significantly, correlated to lordotic correction (r = –0.52, P = 0.06, F = 2.85, Y = –0.004X–0.026). MDH change was the least correlated to lordotic change (r = 0.47, P = 0.09, F = 2.36, Y = 0.003X–0.010). See Table 3 for a summary of these results.

**Table 3:** Linear correlation and regression analysis results Abbreviations: ADH, anterior disc height; MDH, middle disc height; PDH, posterior disc height *Positive values denote a reduction in disc height; negative values denote an increase in disc height.

Comparison	Models Included in Comparison	Pearson’s Correlation Coefficient*	P Value
Pre-correction PDH vs lordotic correction achieved	Horizontally printed models with PolyFlex: models 1-3, 8, 10	0.56	0.12
Change in PDH vs lordotic correction	Models 1-10	0.92	<0.001
Change in MDH vs lordotic correction	Models 1-10	0.47	0.09
Change in ADH vs lordotic correction	Models 1-10	-0.52	0.06

Material comparisons demonstrated that PolyFlex and soft polylactic acid performed similarly, but thermoplastic polyurethane resulted in significantly less lordotic correction. Comparisons were also made between models printed with variable bony and soft tissue densities. Models with higher bone densities achieved a lordotic correction that was much greater than the cadaveric comparison data [unpublished data]. Models printed either vertically (models 4-6), or with low bone density and high soft tissue density (model 10), appeared to perform the most similarly to the cadaveric tissue [unpublished data].

## Discussion

**Figure 4:**
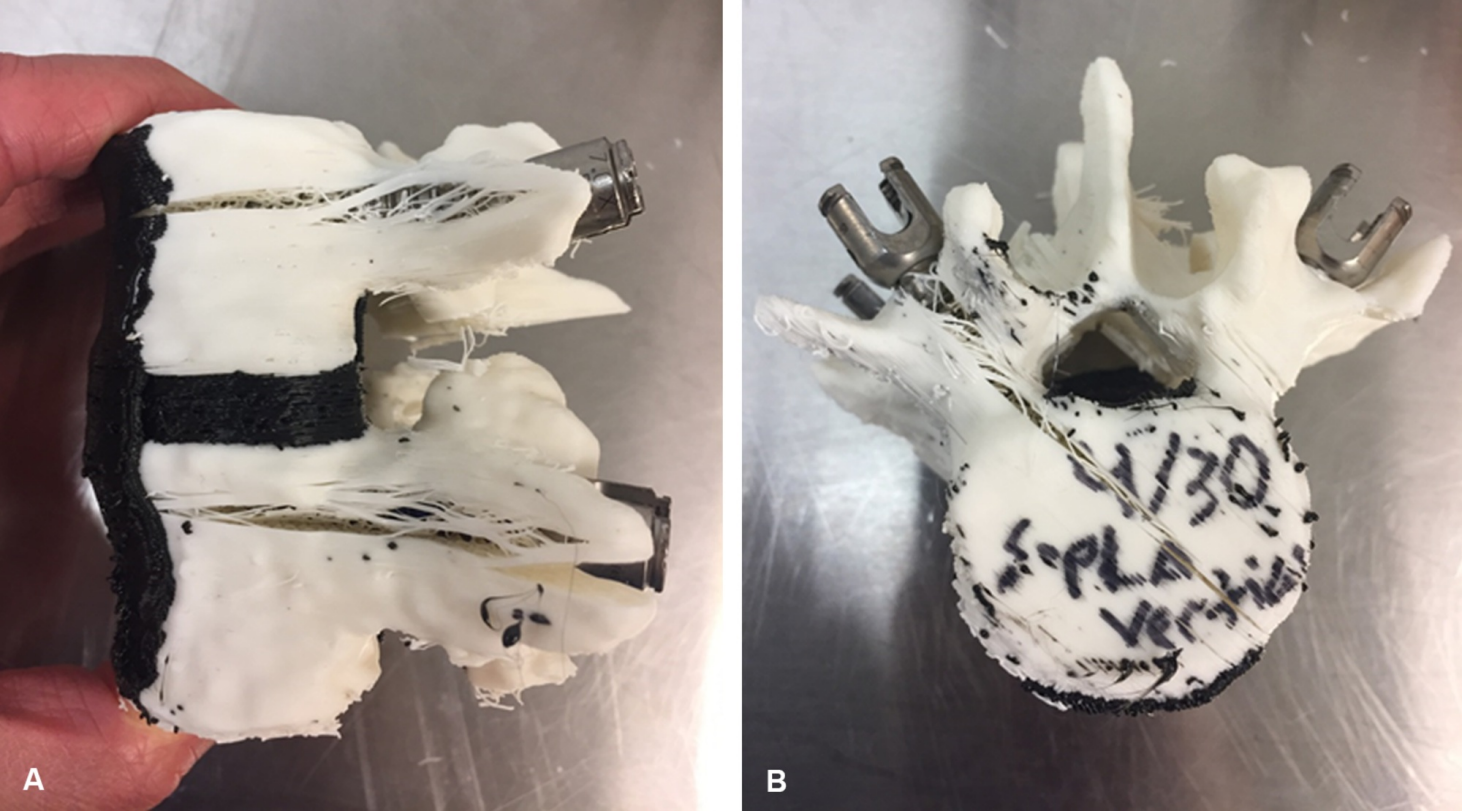
Photographs of (A) a horizontally printed model and (B) a vertically printed model after pedicle failure. Note how the horizontal model fails in a different plane than the vertical model. The vertically printed model fractures in a plane parallel to the compressive force between pedicle screws, therefore resulting in earlier pedicle failure and smaller lordotic correction.* Used with permission from Barrow Neurological Institute, Phoenix, Arizona.*

Previous studies assessing the vertebral bodies of the Barrow Biomimetic Spine model demonstrated that print orientation has a significant effect on the biomechanical performance of pedicle screws in 3D-printed vertebral body models [unpublished data]. Specifically, horizontally printed models had significantly greater strength on axial pullout testing than vertically printed models. Interestingly, the print orientation of the L3-L5 synthetic models tested in this study also had a significant impact on model performance. Examination of the models after lordotic correction revealed the probable reason for vertically printed models achieving less lordotic correction than horizontally printed models; the orientation of the 3D-printed layers in the vertical models is parallel to the vector of compressive force during lordotic correction. This means that models printed vertically will demonstrate pedicle fracture and pedicle screw failure with much less compressive force than in the horizontally printed models, resulting in less lordotic correction. Figures 4A-4B demonstrate horizontally and vertically printed models after being compressed to the point of pedicle fracture, demonstrating how the vertically printed models fracture differently than the horizontally printed models. Interestingly, the horizontally printed models were found to generate too much lordotic correction compared to similar cadaveric models. The vertically printed models failed much sooner during a lordotic correction, and therefore achieved a much smaller percent error.

Material selection also appeared to play a significant role in model performance, with thermoplastic polyurethane performing more poorly than PolyFlex or soft polylactic acid. The Shore hardness of thermoplastic polyurethane is 94A, whereas PolyFlex and soft polylactic acid each have estimated values closer to 90A. The increased hardness of the thermoplastic polyurethane likely explains why a much smaller lordotic correction was achieved with its use as a soft tissue surrogate. It is also important to note that the printed density of the bone and soft tissue model components impacted performance. As would be predicted, less-dense bone models appear to achieve less correction, with the least correction occurring for models printed with low bone density and high soft tissue density. This makes sense considering that the less-dense bone in these models is compressed across a denser and more rigid disc space, resulting in pedicle failure much earlier in the lordotic correction. This is an important finding because it demonstrates that the synthetic models perform in a predictable and reliable fashion. It is also important to note that the model printed with the least-dense bony material and the densest soft tissue (model 10) was the best performing horizontally printed model. This finding is consistent with the better performance identified in vertically printed models, which also demonstrated earlier pedicle failure during lordotic correction.

The synthetic models also demonstrated predictable lordotic correction based on pre-correction PDH measurements; this finding demonstrates that the synthetic spine models perform analogously to the comparison cadaveric tissue. Furthermore, the synthetic models that achieved greater degrees of correction demonstrated a greater expansion of the ADH, suggesting a dynamic shifting of the fulcrum of sagittal rotation during lordotic correction from the anterior longitudinal ligament to the posterior longitudinal ligament once the PDH is maximally compressed. This is an important finding because it demonstrates that the synthetic models are not only capable of achieving the same lordotic correction as the cadaveric tissue, but are also achieving this lordotic correction through a similar means of dynamic compression of the PDH followed by expansion of the ADH.

## Conclusions

The 3D-printed synthetic spine models described above demonstrated predictable and reliable performance in a lordotic correction test based on their respective material qualities and printed densities. The print variables tested furthermore demonstrated that this model is capable of achieving high biomechanical fidelity to cadaveric tissue when subjected to the same lordotic correction test after Schwab grade 2 osteotomies. Future studies will assess the performance of this model as compared to cadaveric tissue when posterior ligamentous structures are added, and when disc-disrupting procedures such as microdiscectomy or interbody graft placements are performed.
